# Grand Challenges to Launching an Ideal Platform for Publishing Microbe-Insect Symbiosis Studies

**DOI:** 10.3389/fmicb.2019.02542

**Published:** 2019-11-11

**Authors:** Takema Fukatsu

**Affiliations:** National Institute of Advanced Industrial Science and Technology (AIST), Tsukuba, Japan

**Keywords:** symbiosis, microbe, insect, publishing, grand challenge

Symbiotic microorganisms are omnipresent in nature, ubiquitously associated with animals, plants, fungi, protists, and all other life forms including humans, ranging from having parasitic through commensalistic to mutualistic associations, and affecting every biological aspect of innumerable organisms living on earth (McFall-Ngai et al., 2013; Webster, 2014). Insects represent the majority of macroscopic biodiversity described thus far (Grimaldi and Engel, 2005), and their ubiquitous interactions with microbes underpin their diversity and adaptability in the ecosystem (Bourtzis and Miller, 2003; Zchori-Fein and Bourtzis, 2011).

Studies on microbe-insect symbioses have a long history. During the early to mid twentieth century, microbe-insect symbioses were regarded as a focal research area in microbiology. Before the era of molecular biology, high-resolution light microscopy developed by Carl Zeiss and other optic companies was the cutting-edge technology in biology. Using high-quality microscopes, a number of German and other European microbiologists enthusiastically surveyed diverse insects, terrestrial arthropods, and other organisms for their internal microbiota. The enormous number of microscopic observations were compiled by the outstanding German microbiologist, Paul Buchner (Sapp, 2002), in the monumental book “*Endosymbiosis of Animals with Plant Microorganisms*” (Buchner, 1965). Subsequently, however, advancements in this research area were for decades very slow, mainly because of the general uncultivability of the symbiotic microorganisms—researchers could observe some bacteria residing in and associated with insect cells and tissues cytologically but were unable to characterize or even identify the microbes. In the 1980s, the invention of PCR and the development of DNA sequencing technologies brought about an epoch-making breakthrough in microbiology—environmental microorganisms became identifiable without cultivation on the basis of 16S rRNA gene sequencing. I remember that, in 1989, just before I began my graduate work at the University of Tokyo in the lab of Hajime Ishikawa, who was among the pioneers of the molecular and genomic aspects of aphid endosymbiotic bacteria (Fukatsu, 2006), the first 16S-based molecular phylogenetic identification of an uncultivable aphid endosymbiont [later named *Buchnera aphidicola* (Munson et al., 1991)] was published by Paul Baumann's group at the University of California, Davis (Unterman et al., 1989). The idea that this extremely specialized insect symbiont is allied to *Escherichia coli* was a big surprise at that time and served as strong motivation for the field to reach a better understanding of the fastidious microorganisms that are closely allied with insects. In 2000, when I had already started to run my own lab, Ishikawa's group determined the first complete genome of an uncultivable microbial mutualist of the pea aphid, *Buchnera aphidicola* (Shigenobu et al., 2000), which opened a new era of powerful genomic approaches to microbe-insect symbiosis studies. At that time, Sanger-based DNA sequencing technology was so time-, labor- and cost-intensive that the microbial genomics was not easily accessible for the majority of individual microbiologists. However, from 2007 onward, high-throughput DNA sequencing technologies, initially 454 and Solexa and then Illumina, PacBio, Nanopore, and others, became available, which propelled the explosive accumulation of microbial genome data. I was amazed to see interesting insect genomes coming up one after another from Nancy Moran's group at the University of Arizona (McCutcheon and Moran, 2007; McCutcheon et al., 2009; Moran et al., 2009), and I soon also jumped into the excitement. Sequencing insect symbiont genomes is like opening treasure boxes, uncovering a variety of astonishing evolutionary aspects such as initial massive accumulation of junk DNA elements (Wu et al., 2004; Toh et al., 2006) and subsequent size reduction, often approaching organelle-like sizes (Nakabachi et al., 2006; Pérez-Brocal et al., 2006), catastrophic genome erosion finally leading to symbiont replacements (Campbell et al., 2017; Matsuura et al., 2018), metabolic complementarity between co-evolving reduced symbiont genomes (McCutcheon and Moran, 2007, 2010), extremely tiny symbiont genomes streamlined for specific biological functions (Anbutsu et al., 2017; Salem et al., 2017), dynamic lateral gene transfer and functional fusion across symbiont and host (Dunning-Hotopp et al., 2007; Husnik et al., 2013), and others. The number of publications on microbe-insect symbioses from 1985 to 2018 can be seen in Figure 1, in which the above-mentioned historical trajectory and development are impressively illustrated in relation to the technological innovations.

**Figure 1:**
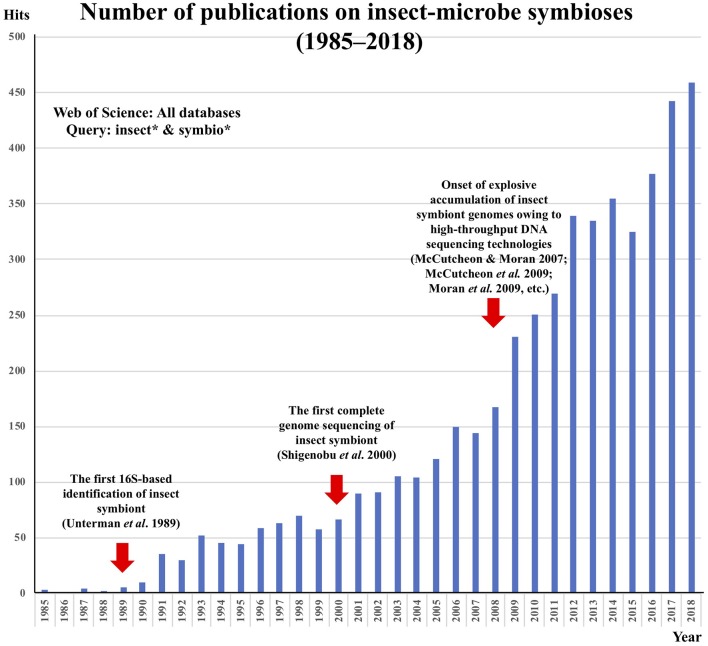
Number of publications on microbe-insect symbioses from 1985 to 2018.

Inherently, studies on symbiosis are destined to be interdisciplinary, encompassing the fields of ecology, evolution, genomics, and cellular and molecular biology. Reflecting this, despite the large number of scientific journals published in the world, few focus on the publication of microbe-insect symbiosis studies. To my knowledge, *Symbiosis* (https://link.springer.com/journal/13199) is the only journal whose mission is to publish papers on animal-microbe, plant-microbe, microbe-microbe, and other forms of symbiotic associations. *Molecular Plant-Microbe Interactions* (https://apsjournals.apsnet.org/loi/mpmi) publishes a considerable number of symbiosis-related papers, though restricted to plant-microbe symbioses. The Invertebrate Microbiology section of *Applied and Environmental Microbiology* (https://aem.asm.org/) and the Microbe-Microbe and Microbe-Host Interactions field of *the ISME Journal* (https://www.nature.com/ismej/) are the suitable outlets for papers on microbe-insect symbiotic associations. *Journal of Invertebrate Pathology* does publish papers on insect symbionts, but the journal mainly focuses on parasites and pathogens (https://www.journals.elsevier.com/journal-of-invertebrate-pathology). *Environmental Entomology* (https://academic.oup.com/ee) highlights *Insect-Symbiont Interactions* as a subject area.

Personally, it has been a challenging task for me to find suitable journals in which to publish my own microbe-insect symbiosis studies. For example, dating back to the early 1990s for my master's thesis at the University of Tokyo, I wrote up three papers on a variety of bacterial and fungal symbionts of aphids. I finally published them in entomological journals, *Insect Biochemistry and Molecular Biology* and *Journal of Insect Physiology* (Fukatsu and Ishikawa, 1992a,b,c) but could not help feeling that those were not actually a perfect fit. The absence of suitable outlets for insect symbiosis studies has been a long-lasting frustration for me, and this frustration must be shared generally by those who are working on such interdisciplinary research fields related to symbiosis.

In this context, it was a laudable decision of *Frontiers in Microbiology* to launch the Microbial Symbioses section in 2013. Since then, the Microbial Symbioses section has served as a home to numerous innovative, high-quality research studies, and commentaries on symbiosis. As a newly-appointed Specialty Chief Editor, I am highly motivated to build up an ideal platform for publishing microbe-insect symbiosis studies in the Microbial Symbioses section of *Frontiers in Microbiology*. To that end, I have contacted the world's most qualified experts who are actively working on microbe-insect symbiotic associations and interactions and have organized a launching Editorial Board for microbe-insect symbioses consisting of 14 Associate Editors as of September 2019. To my knowledge, no other scientific journals specialize in the publication of microbe-insect symbiosis studies. Though not as a journal but as a part of the journal section, I am aiming at manifesting such a publishing body of high quality and visibility, which offers prompt handling, fair and robust evaluation, and qualified publication of submitted manuscripts covering diverse aspects of microbe-insect symbiotic associations and interactions. The open access policy of *Frontiers in Microbiology*, together with the notable impact factor of 4.259, provides a good reason for consideration. I look forward to the very best works on microbe-insect symbioses being submitted to *Frontiers in Microbiology*.

## Author Contributions

The author confirms being the sole contributor of this work and has approved it for publication.

### Conflict of Interest

The author declares that the research was conducted in the absence of any commercial or financial relationships that could be construed as a potential conflict of interest. The reviewer JM declared a past collaboration with the author to the handling editor.
